# Extensive Lupus Vulgaris Mimicking Chromoblastomycosis

**DOI:** 10.4269/ajtmh.24-0165

**Published:** 2024-07-02

**Authors:** Priyansh Gupta, Biswanath Behera, Madhusmita Sethy, Vishal Thakur

**Affiliations:** ^1^ Department of Dermatology and Venereology, All India Institute of Medical Sciences, Bhubaneswar, India;; ^2^ Department of Pathology, All India Institute of Medical Sciences, Bhubaneswar, India

A 28-year-old male presented with multiple raised lesions of 1 year’s duration over the right foot, leg, and knee. He denied any history of trauma before the onset of the lesions. Cutaneous examination showed multiple grayish papules and plaques of variable size ranging from 1 to 10 cm, with a verrucous surface and overlying hemorrhagic crust over a few lesions (Figure 1) and intervening areas of scarring. Clinical differentials of cicatricial chromoblastomycosis and lupus vulgaris (LV) were considered. The potassium hydroxide (KOH) mount from the hemorrhagic crust did not reveal any fungal elements. Histopathological examination of the lesion showed hyperkeratotic epidermis with hypergranulosis and acanthosis and epithelioid granulomas with foreign body–type giant cells and diffuse dense inflammation composed of lymphocytes, plasma cells, and few neutrophils and eosinophils in the dermis (Supplemental Figures 1 and 2). Gomori’s methenamine silver stain and Ziehl-Neelsen stain were negative. There was no growth on tissue fungal or bacterial culture. Tissue polymerase chain reaction for tuberculous bacilli was negative. QuantiFERON-TB gold assay was positive. In view of the positive TB gold assay, the presence of epithelioid granulomas, and the absence of fungal hyphae on KOH and histopathology, a diagnosis of LV was considered. The patient was started on antitubercular therapy (ATT) per the national program (Index-TB guidelines),^1^ and he showed a response within 6 weeks and almost complete clearing of the lesions after 6 months of therapy (Supplemental Figure 3).

**Figure 1.**
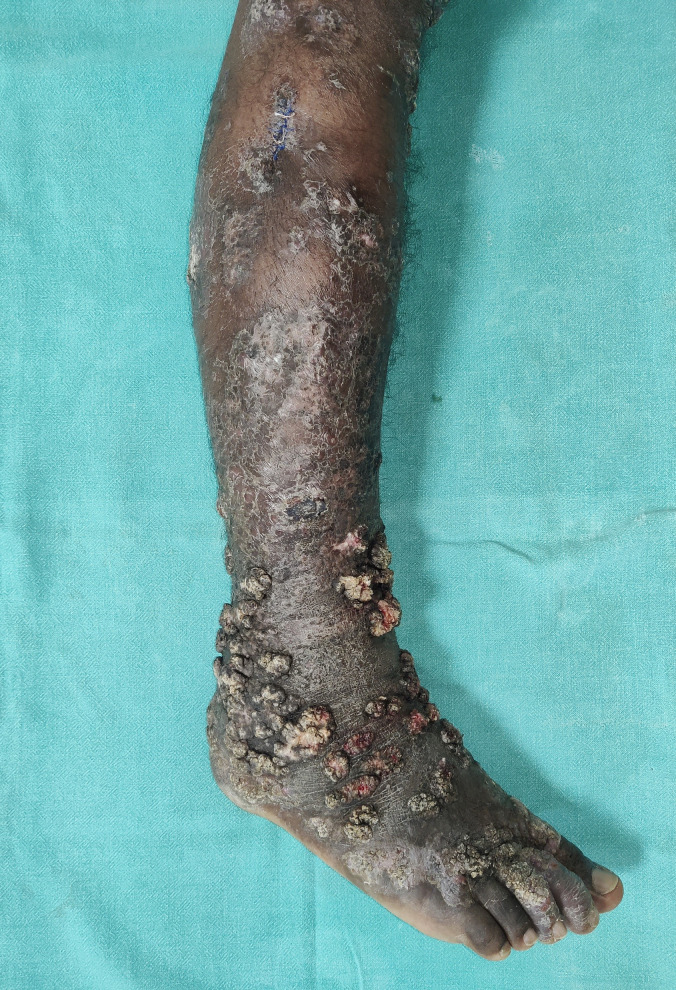
Clinical image of the lesions over the right lower limb showing multiple pigmented papules and plaques with surface verrucosities over a few lesions, hemorrhagic crust, and intervening areas of scarring.

Verrucous LV or tuberculosis verrucosa cutis–like LV is a rare variant of LV. The diagnosis of LV is usually based on a combination of clinical, histological, and microbiological findings. The paucibacillary nature of LV makes detection of mycobacteria in skin lesions very difficult, and cultures are often negative. Chromoblastomycosis, a close mimicker of LV especially when lesions are verrucous in appearance, usually shows the presence of copper penny bodies on KOH or histopathology, a positive fungal culture, and a good response to antifungal treatment. In our case, clinical features supported by histopathologic findings, positive QuantiFERON TB gold assay, and complete therapeutic response to ATT confirmed the diagnosis of LV.

## Supplemental Materials

10.4269/ajtmh.24-0165 Supplemental Materials
