# Supplementary figures and images for: Extensive Lupus Vulgaris Mimicking Chromoblastomycosis
Source: Am J Trop Med Hyg. 2024 Jul 2;111(3):452. doi: 10.4269/ajtmh.24-0165 (PMC11376163; doi:10.4269/ajtmh.24-0165)

Supplemental Fig1

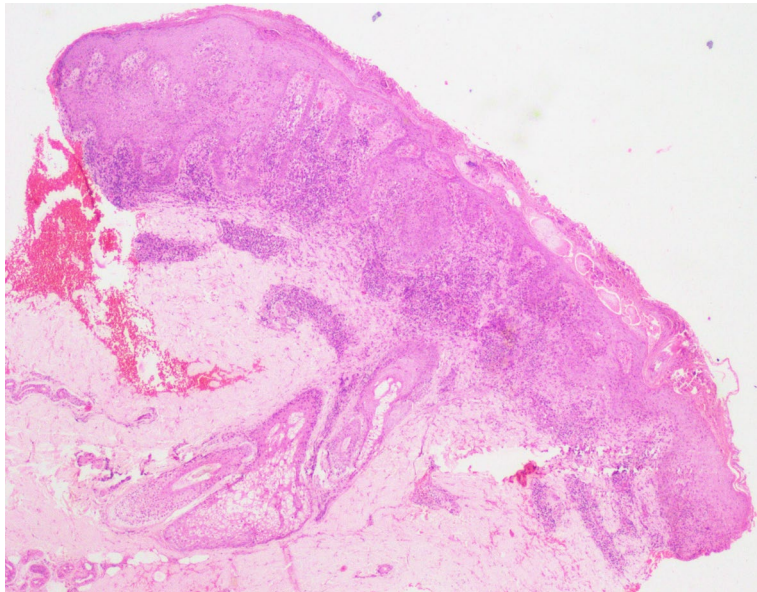

Supplemental Fig2

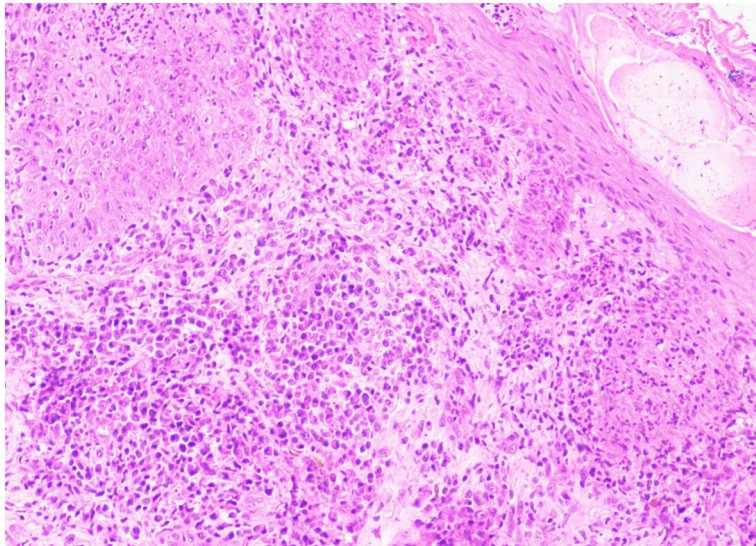

Supplemental Fig3

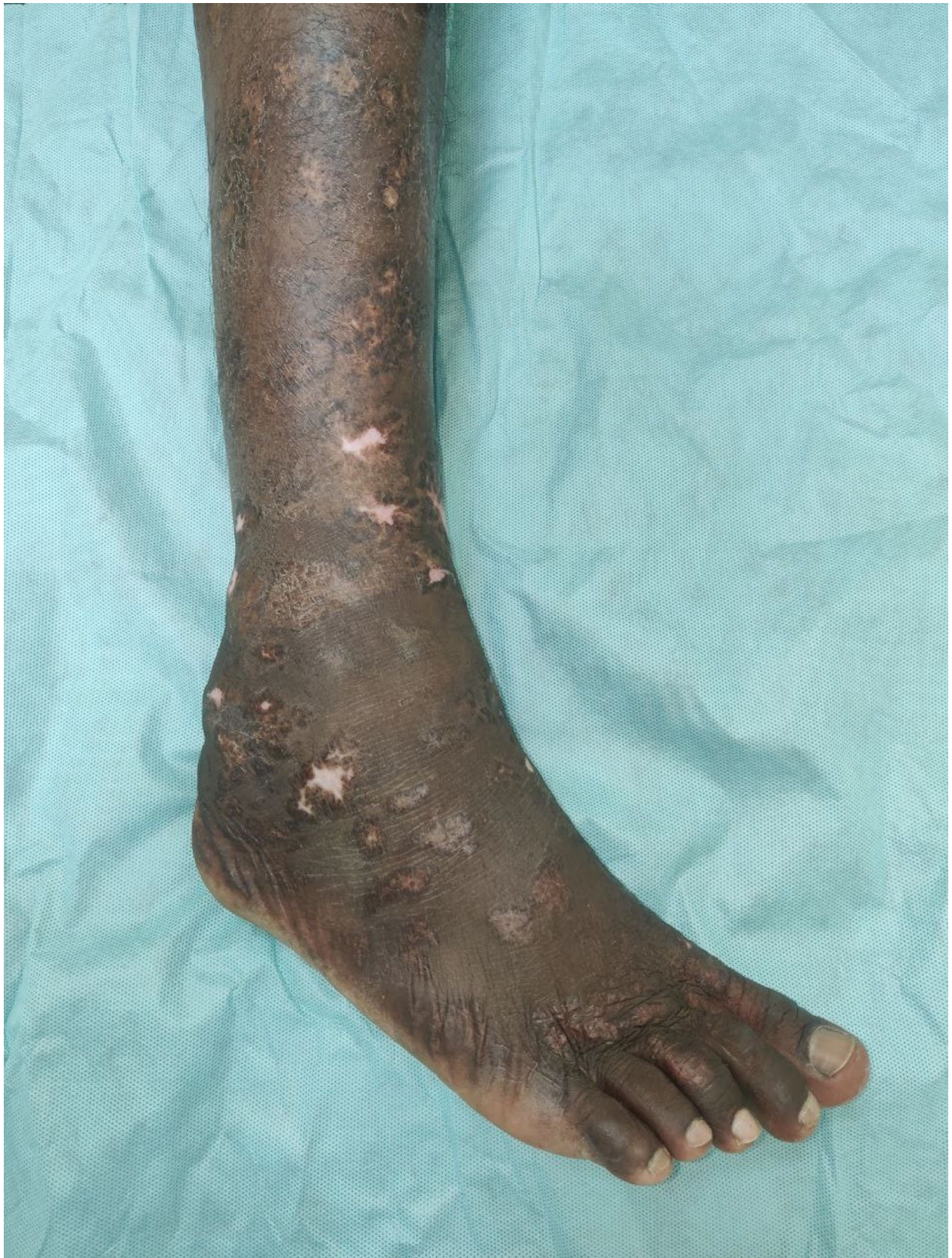

Supplement: Supplemental Materials [file tpmd240165.SD1.pdf]
